# The long-term effect of biochar application to *Vitis vinifera* L. reduces fibrous and pioneer root production and increases their turnover rate in the upper soil layers

**DOI:** 10.3389/fpls.2024.1384065

**Published:** 2024-10-17

**Authors:** Peter Beatrice, Michele Dalle Fratte, Silvia Baronti, Alessio Miali, Lorenzo Genesio, Francesco Primo Vaccari, Bruno E. L. Cerabolini, Antonio Montagnoli

**Affiliations:** ^1^ Laboratory of Environmental and Applied Botany, Department of Biotechnology and Life Sciences, University of Insubria, Varese, Italy; ^2^ Unit of Plant Ecology and Phytogeography, Department of Biotechnology and Life Sciences, University of Insubria, Varese, Italy; ^3^ Institute of BioEconomy, National Research Council, Florence, Italy

**Keywords:** fine root, functional traits, grapevine, soil carbon, specific root length, soil water content, climate change

## Abstract

Fibrous and pioneer roots are essential in the uptake and transport of water and nutrients from the soil. Their dynamic may be influenced by the changing of soil physicochemical properties due to the addition of biochar, which, in turn, has been shown to improve plant growth and productivity in the short term. However, the long-term effects of biochar application on root dynamics are still widely unknown. In this study, we aimed to investigate the long-term effects of biochar application on grapevine fibrous and pioneer root dynamics and morphological traits in relation to soil characteristics. To this aim, grapevine plants amended in 2009 and 2010 respectively with one and two doses of biochar, were analyzed in their fibrous and pioneer root production and turnover rate, standing biomass, length, and specific root length, over two growing seasons. Our findings demonstrate that in the long term, biochar application significantly increased soil pH, nutrient availability, and water-holding capacity causing a decrease in the production of fibrous and pioneer roots which is reflected in a reduction of the root web characterized though by a higher turnover rate. Furthermore, we observed that these root morpho-dynamical changes were of higher magnitude in the upper soil layers (0-20 cm) and, at least in the long term, with no significant difference between the two doses. These results suggest that in the long term, biochar can be a powerful tool for improving soil quality, which in turn lowers carbon-cost investment toward the root production and maintenance of a reduced root web that might be directed into grapevine growth and productivity. Such effects shed some light on the root plastic and functional adaptation to modified soil conditions facilitated by the long-term application of biochar, which can be used for implementing adaptive agricultural practices to face the current climate change in a frame of sustainable agricultural policies.

## 1 Introduction

Fine roots are essential for the uptake and transport of water and nutrients from the soil to the plant tissues (Sattelmacher et al., 1990). They respond quickly to environmental cues, such as resource availability and soil bulk density, through a species-specific modulation of traits that allow for optimal use of underground resources (McCormack et al., 2015; Razaq et al., 2017; Ma et al., 2018; Montagnoli et al., 2018, 2019; Ola et al., 2018). Generally, the modulation of fine root traits involves the production of roots with different forms and functions, namely fibrous and pioneer roots (Guo et al., 2004). Pioneer roots are characterized by a relatively coarse diameter, limited mycorrhizal colonization, and a long average life expectation as they undergo secondary growth (Polverigiani et al., 2011). These roots represent the framework of an expanding root system in the vertical and horizontal direction, mainly functioning in nutrient and water transport and acting as storage organs for carbohydrates and nutrients (Polverigiani et al., 2011). Fibrous roots have a smaller diameter and do not undergo secondary growth (Xia et al., 2010); hence, they have lower carbon costs production (Ostonen et al., 2007) and a fast turnover rate (Joslin et al., 2006) living rarely more than one year (Wells and Eissenstat, 2001). Being often colonized by mycorrhizal fungi, fibrous roots mainly function in water and nutrient absorption from the soil (Polverigiani et al., 2011). Fibrous and pioneer roots differ in their morpho-physiological response to variations in soil conditions and environmental stresses, including moisture and nutrient deficiency (Polverigiani et al., 2011; Montagnoli et al., 2021), freeze-thaw cycles (Yin et al., 2017), and excavation disturbance (Nakahata, 2020), and thus their response may vary following the use of soil amendments.

Biochar, produced by the pyrolysis or gasification of organic matter, has gained interest as a potential soil fertility enhancer, representing at the same time a possible solution for waste biomass valorization and for climate change mitigation by sequestering carbon in the soil (Ceriani et al., 2023). Biochar has been shown to influence soil physicochemical properties (Hardie et al., 2014), resulting in improved plant growth (Polzella et al., 2020; Simiele et al., 2022a, 2022b) and productivity (Genesio et al., 2015). The mechanisms behind the soil improvement with biochar are likely multifaceted and may include the increase in soil organic matter (Laird, 2008), changes in nutrient cycling (Biederman and Stanley Harpole, 2013), and alterations in soil structure (Hardie et al., 2014). However, the biochar effects are variable, depending on the feedstock properties (Ceriani et al., 2024), pyrolytic temperatures, and heating rate used for its production (Rogovska et al., 2012), as well as on the soil type it is applied to (Beatrice et al., 2023) and application dose (Laghari et al., 2015). Regarding the latter, numerous studies found that high application rates of biochar don’t necessarily ensure improved plant growth with respect to lower rates, suggesting the existence of a saturation point (Genesio et al., 2015), beyond which, an excessive biochar amendment dose can even lead to adverse effects and decreased plant growth (Laghari et al., 2015). Since roots represent the first organ in contact between plants and biochar particles, their production may be altered by biochar application, in turn affecting plant performance (Montagnoli et al., 2021). In a recent meta-analysis, Xiang et al. (2017) concluded that biochar application affects numerous morphological root traits such as biomass, volume, surface area, length, number of root tips, and diameter. Since fine roots play a crucial role in adsorbing and transporting nutrients and water from the soil (Razaq et al., 2017; Montagnoli et al., 2021) their modulation can change following biochar application. Given the economic and cultural significance of grapevine cultivation worldwide, understanding how biochar influences grapevine growth and root dynamics is crucial for developing sustainable viticulture practices that optimize productivity and soil health in the long term. Numerous studies, conducted on the same biochar-amended vineyard in the period 2009-2019, showed positive effects in multiple physicochemical characteristics of the soil. Among these, increased pH (Maienza et al., 2017; Giagnoni et al., 2019; Baronti et al., 2022; Idbella et al., 2024), increased water content (Baronti et al., 2014), increased organic carbon (Giagnoni et al., 2019; Idbella et al., 2024), and decreased bulk density (Baronti et al., 2014, 2022; Idbella et al., 2024) was always observed in biochar-treated soil, even after 10 years from the amendment (**Supplementary Table S1**). Research on root system responses to biochar is less common, primarily due to the challenges involved in studying underground plant organs. However, grapevine roots have demonstrated a positive reaction to the beneficial conditions that biochar creates in the soil (Montagnoli et al., 2021). A study on short-term biochar-treated grapevine plants observed an increase of fine root biomass after 3-4 months from the treatment, determined by the stimulation of the radial root growth (root diameter) rather than the longitudinal growth (root elongation) (Amendola et al., 2017). This response could be related to the improved water and nutrient availability in the soil so that the cost-benefit balance favored the improvement of the transport system instead of increasing soil exploration. For the same reason, in summer, grapevine plants grown on biochar-treated soils have usually a lower number of fibrous roots (Montagnoli et al., 2021). Therefore, biochar-induced shifts in allocation between fibrous and pioneer roots may only occur at specific times of the year (Comas et al., 2005; Montagnoli et al., 2021). However, despite many studies focusing on the short term, further research is still needed to understand the specific mechanisms underlying the long-term effects of biochar addition (Gul et al., 2015), especially on fibrous and pioneer root dynamics. For example, seven years after biochar application, rice productivity was still enhanced through the regulation of root development (Liu et al., 2021). After ten years, Baronti et al. (2022) found that biochar amendment led to a general reduction of the grapevine fine root standing biomass and length in the upper 20 cm soil layers, probably due to the lower need for fine root foraging. As biochar application to soil is an irreversible practice, the need to provide better explanations for changes in root dynamics as biochar undergoes aging is urgent (Singh and Cowie, 2014; Baronti et al., 2022). The impact of biochar on soil biology and physicochemical properties is likely different from that observable in the short term since its properties could change over time (Hardy et al., 2019; Idbella et al., 2024). Recent studies highlighted that in the long term, biochar can affect soil chemistry, biochemistry, and microbiota (Maienza et al., 2017, 2023; Hardy et al., 2019), but very few studies are currently available about the long-term effects on fine roots dynamics (Baronti et al., 2022; Liu et al., 2021). However, this knowledge can only be obtained with long-term studies.

We hypothesized that, in the long-term, biochar application (*i*) enhances soil physicochemical properties increasing water and nutrient availability, and in turn (*ii*) decreases and increases respectively the production and turnover rate of pioneers and fibrous roots that is reflected in (*iii*) a lowering of the root length and biomass development. Moreover, we hypothesized that these changes are more pronounced (*iv*) in the higher biochar dose treatment and (*v*) in the upper soil layers where biochar is incorporated into the soil and is more abundant. To test our multiple hypothesis, we investigated the long-term effects of biochar application on soil physicochemical properties coupled with fibrous and pioneer root dynamics (production and turnover rate) and morpho-functional traits (length, biomass, and specific root length - SRL) on grapevine plants (*Vitis vinifera* L.) ten years after the application of two different biochar doses. Our objective was to understand how *V. vinifera* plants modify the growth of their fine root systems to adjust to the soil physicochemical modification due to the long-term biochar effect.

## 2 Materials and methods

### 2.1 Site characteristics and experimental design

The study site is the vineyard “La Braccesca Estate” (Marchesi Antinori srl), located in Montepulciano (Tuscany, Central Italy, 43°10’15” N, 11°57’43” E, 290 m a.s.l.), and planted in 1995 with *Vitis vinifera* L. cv. *merlot*. Plants were organized on a single curtain trellis system with a plant spacing of 0.8 m within the row and 2.5 m between rows. Based on the data of the nearby Cortona meteorological station (43°16’05” N, 11°59’46” E, 427 m a.s.l., https://www.sir.toscana.it/, ID TOS01000751) over the study period (2009-2020), the climate of the study area is typically Mediterranean, characterized by a mean annual temperature of 14.6°C and cumulated precipitations of 788.5 mm, the hottest and driest months being July (31.3°C and 34.2 mm) and August (31.0°C and 46.1 mm), which represent the summer drought period. The coldest months are January and February (respectively, 2.3 and 2.6°C), which are characterized by an average cumulated precipitation (respectively, 54.5 and 61.7 mm), while there are two rainy seasons in spring and summer, respectively May (77.7 mm) and November (105.8 mm). The soil has developed on lacustrine sediments, it is acidic, shallow, sandy-clay-loam textured (National Soil Survey Handbook, 2005), and highly compacted below the depth of 40 cm. The soil physicochemical properties of the experimental vineyard were described in detail by (Baronti et al., 2014).

A randomized plot experiment with three treatments and five replicates (total number of plots = 15) was started in 2009 (Baronti et al., 2014, 2022). Each plot had a surface area of 225 m^2^ (7.5 m in width and 30 m in length), including four vineyard rows and three inter-rows. The treatments corresponded to control (C; n = 5, no biochar application), single-dose biochar (B; n = 5, application of biochar at a rate of 16.5 dry t ha^−1^ in 2009), double-dose biochar (BB; n = 5, application of biochar at a rate of 16.5 dry t ha^−1^ in 2009 and 2010).

The biochar used in the experiment was obtained by pyrolysis at 500°C of orchard pruning residues (Romagna Carbone, Bagnacavallo, Ravenna, Italy). The physicochemical properties of the biochar were illustrated in detail by (Baronti et al., 2014), the main one being 77.8% total carbon, 0.91% total nitrogen, 101 cmol_C_ kg^−1^ cation exchange capacity, and 2722 mm^3^ g^−1^ porosity. The biochar was crushed into particles smaller than 5 cm in diameter and mechanically mixed to the inter-row soil of the vineyard using a spreader and a chisel plow tiller, reaching a soil depth of 30 cm.

Since the start of the experiment, all treatments have been managed without irrigation and applying a dose of 120 kg ha^−1^ of inorganic fertilizer (NPK 15-0-26) twice a year (240 kg ha^−1^ per year). To control weed and grass growth, the vineyard follows a three-year alternate management practice. Each year, one inter-row is managed, while the following two are left unmanaged. The farm works on the managed inter-row using a rototiller that affects the 0-20 cm soil layer. The two adjacent unmanaged inter-rows are left covered with spontaneous grass, which is mowed twice a year, respectively in late spring and in summer.

### 2.2 Grape yield

In September 2019, grapes from 5 plants for each plot were hand-harvested and weighed; the obtained means are shown in the results section.

### 2.3 Fine roots sampling, processing, and measurement

We used the soil core sampling method (Vogt and Persson, 1991) to quantify the fibrous and pioneer root traits. Within four plots per treatment and at four different sampling dates, two soil cores with a diameter of 4 cm and a length of 40 cm were collected at 50 cm from the plant stem using a motor-driven portable core sampler adapted from Ponder and Alley, 1997. The soil cores were collected through the 2019 growing season (May 16th, June 28th, and September 19th) and in spring 2020 (April 15^th^), covering different grapevine phenological stages, respectively: main flowering, end of flowering, fruit ripening, and budburst. Due to logistical constraints, samples were collected only on four out of five replicates for each treatment. The 40 cm soil cores were divided into 4 soil layers of 10 cm each, stored in plastic bags at 4°C, and processed within 20 days from collection. To extract the roots from the soil, (*i*) each sample was placed in a nylon bag with a 300 μm mesh; (*ii*) each nylon bag was washed with cold water into a washing machine to let the soil sieve out and retain roots and stones within the nylon bag (adapted from Benjamin and Nielsen, 2004); (*iii*) finally, fine roots were visually separated from stones and soil remains with the aid of a stereomicroscope (Nikon SMZ 800). Once separated from the soil, roots were visually divided into two main groups, grapevine roots and other species roots, taking advantage of the different structure and texture of woody plant species roots compared to herbaceous ones. Subsequently, grapevine roots were visually segregated into live (biomass) and dead (necromass) roots according to differences in color, texture, turgor, and shape (Vogt and Persson, 1991).

Both live and dead roots were scanned submerged in water at 800 dpi using a calibrated flatbed scanner coupled to a transparency unit for high-resolution image acquisitions (Epson Expression 10,000 XL). The images were subsequently analyzed with the WinRhizo Pro V. 2007d software (Regent Instruments Inc. Quebec, Canada) to measure fine root length (RL) and volume (RV) for different diameter classes as well as for live and dead roots. Live and dead roots were separately oven-dried at 70°C until constant weight to obtain the total root biomass (RB) and necromass (RN) values. Roots were considered pioneer roots (Pion) in the diameter range 0.35-1.30 mm and fibrous roots (Fibr) in the range 0.15-0.30 mm based on diameter categories previously observed in *Vitis vinifera* through a rhizobox approach by (Montagnoli et al., 2021). Roots exceeding 1.30 mm in diameter were excluded from this study. The root volume was multiplied by the tissue density (RV to RB ratio) previously measured on a representative aliquot of roots of both diameter classes (fibrous and pioneer roots) and each treatment (C, B, BB) to calculate the root biomass for each diameter class. The specific root length (SRL) was calculated as the RL to RB ratio.

Dead roots data were used only for calculating the annual root production and turnover rates. The annual root production was estimated using the minimum–maximum method procedure, which allows us to consider the multimodal seasonal pattern characterized by seasonal variation of root biomass dynamics (Edwards and Harris, 1977). This method calculates and sums only significant differences between seasonal minimum and maximum dry root mass (RB and RN) (McClaugherty et al., 1982). This method yielded realistic results in *Fagus-Quercus* mixed forests (Hertel and Leuschner, 2002) but was also used successfully with grapevine (Amendola et al., 2017). The root turnover rates were calculated as annual root production (RB and RN) divided by maximum standing root mass (Gill and Jackson, 2000).

### 2.4 Soil sampling and analysis

In July 2020, 11 years after the first biochar application and 10 years after the second biochar application, a soil sample was collected at 0-20 cm depth, after removal of above-ground litter, in three points within each plot to form a representative composite sample for each plot. A total of five samples were collected for each treatment (C, B, and BB). For each sample, we measured the following soil physical and chemical properties: soil pH, measured through a soil:distilled water solution (1:2.5 w:w) stirred for 30 min, settled for 1 h, and finally centrifuged for 10 min.; soil water content, determined volumetrically (Grossman and Reinsch, 2018); soil bulk density, calculated as the dry weight-to-volume ratio; cation exchange capacity (CEC), determined using the NH_4_OAc method; total organic carbon (C_org_), measured using the method of Walkley and Black, 1934; total nitrogen (N_tot_), determined with the CHN-S Flash E1112 elemental analyzer (Thermo Finnigan; standard method ISO 10694); available calcium (Ca_av_), potassium (K_av_), magnesium (Mg_av_), and sodium (Na_av_), determined using an ICP-OES spectrophotometer (Varian Inc., Vista MPX) according to the EPA method 3052 (US-EPA, 1996).

The soil water content (0-20 cm) was also measured at different times, following the root sampling dates as much as possible, during the 2019 growing season (May 16^th^, June 28^th^, and September 26^th^) and in spring 2020 (April 20^th^). Soil water content was measured gravimetrically on 15 soil samples for each treatment (C, B, and BB) at sampling dates (Baronti et al., 2022).

### 2.5 Statistical analysis

All the analyses were done with R software (R Core Team, 2023). All the analyses on root traits at each 10 cm soil depth were carried out using the average trait values of the two soil cores collected within each plot at each sampling date (n = 192 for each root type). The analysis in the upper 40 cm soil depth was based on pooled root trait data calculated from root trait values from each 10 cm soil depth layer at each sampling date, by using the sum for RL and RB, or the mean for SRL. The same data were used to calculate root turnover and production in the upper 40 cm soil depth. To compare the effect of treatment (C, B, BB), root type (fibrous and pioneers) and seasonality (four sampling dates) and their interactions in the upper 40 cm soil depth on root traits (RL, RB, SRL, as well as root production and turnover but without date in this case), we fitted a three-way mixed effect aligned ranks transformation analysis of variance (ART ANOVA) using the function “art” of the package “ARTool” (Kay and Wobbrock, 2016), setting the plot as a random effect. After building for each variable (or their interactions) a linear model from aligned and ranked data, the least square means were computed through the function “emmean” of the package “emmeans” (Lenth et al., 2018) that was used to run pairwise *post-hoc* comparisons adjusted by the Bonferroni p-value adjustment method. The same analysis was done to test the effects of treatment, root type, soil depth (0-10, 10-20, 20-30, 30-40 cm), and their interactions on all root traits. Moreover, to help visualize the seasonal pattern of RL, RB, and SRL, we fitted a local polynomial regression using the function “geom smooth” of the “ggplot2” package (Wickham, 2016). One-way mixed-effects ART ANOVA followed by *post-hoc* comparison based on the Bonferroni p-value adjustment method, considering the plot as a random effect, was used to compare soil physicochemical properties across treatments. The existence of linear relationships between root traits (RL, RB, SRL, root production, and turnover rate) and soil physicochemical properties measured at 0-20 cm soil depth across all treatments was then tested using the function “lmp” of the “lmPerm” package (Wheeler and Torchiano, 2016) based on 9999 randomizations and using the plot as a random factor. Linear relationships were tested considering the average of pooled root trait data calculated from root trait values from the upper 20 cm soil depth layer at each sampling date by using the sum for RL, RB, and root production, or mean for SRL and turnover rate. The same analysis was done to test the existence of linear relationships between RL, RB, SRL from the upper 20 cm soil depth layer, and soil water content measured at each sampling date.

## 3 Results

### 3.1 Grape yield

During the 2019 harvest season, control plants produced 1.65 ± 0.09 kg (mean ± standard error) of grapes, while biochar-treated plants produced significantly more grapes, not different from each other, respectively 2.40 ± 0.23 kg with the single biochar application (B) and 2.48 ± 0.28 kg with the double biochar application (BB).

### 3.2 Soil physicochemical proprieties and nutrient contents

Concerning the soil physicochemical properties, the average soil pH was higher in both the B and BB treatments than the control, showing the highest values in the BB treatment (**Table 1**). The average soil water content and cation exchange capacity (CEC) were higher only in the BB treatment, while the control and B treatments showed no significant differences (**Table 1**). By contrast, the average bulk density was lower in both the B and BB treatments than in the control, showing the lowest value in the BB treatment (**Table 1**).

**Table 1:** Soil physical and chemical properties at 0-20 cm depth in relation to different experimental treatments (control, C; biochar single-dose, B; biochar double-dose, BB).

	(C) Control	(B) Biochar single-dose	(BB) Biochar double-dose
pH	5.83 ± 0.12 **^c^**	6.41 ± 0.05 **^b^**	6.98 ± 0.07 **^a^**
Bulk density (g cm^-3^)	1.64 ± 0.01 **^a^**	1.59 ± 0.00 **^b^**	1.55 ± 0.01 **^c^**
Water content (%)	17.13 ± 0.36 **^b^**	17.50 ± 0.44 **^b^**	20.88 ± 0.39 **^a^**
CEC (meq 100g^-1^)	25.68 ± 0.16 **^b^**	26.14 ± 0.37 **^ab^**	28.10 ± 0.65 **^a^**
C_org_ (%)	1.27 ± 0.06 **^c^**	1.73 ± 0.09 **^b^**	2.31 ± 0.14 **^a^**
N_tot_ (%)	0.44 ± 0.01 **^b^**	0.44 ± 0.01 **^b^**	0.51 ± 0.01 **^a^**
K_av_ (mg K_2_O kg^-1^)	192.90 ± 0.39 **^b^**	193.30 ± 0.52 **^b^**	199.30 ± 0.52 **^a^**
Ca_av_ (mg CaO kg^-1^)	1453.60 ± 0.68 **^b^**	1454.40 ± 1.71 **^ab^**	1463.40 ± 1.97 **^a^**
Mg_av_ (mg MgO kg^-1^)	939.19 ± 1.06 **^b^**	941.10 ± 0.46 **^ab^**	944.80 ± 2.12 **^a^**
Na_av_ (mg Na kg^-1^)	98.05 ± 0.43 **^a^**	97.910 ± 0.33 **^a^**	98.86 ± 0.41 **^a^**

CEC, cation exchange capacity; org, organic; tot, total; av, available.

Each value represents the mean of 5 replicates (n = 5) ± standard error. Letters a, b, and c indicate significant differences (p < 0.05) between the soil treatments.

Regarding nutrient contents, the total organic carbon (C_org_) was higher in both the B and BB treatments with respect to the control, showing the highest values in the BB treatment (**Table 1**). Total nitrogen (N_tot_) and available potassium (K_av_) did not show differences between the control and B treatment, while higher values were observed in the BB treatment (**Table 1**). Available calcium (C_av_) and magnesium (Ma_av_) were higher in the BB treatment than in the control, while the B treatment did not show significant differences (**Table 1**). Available sodium (Na_av_) showed no differences between the control and the treatments (**Table 1**).

### 3.3 Relation between fine root traits and soil properties

**Figure 1:**
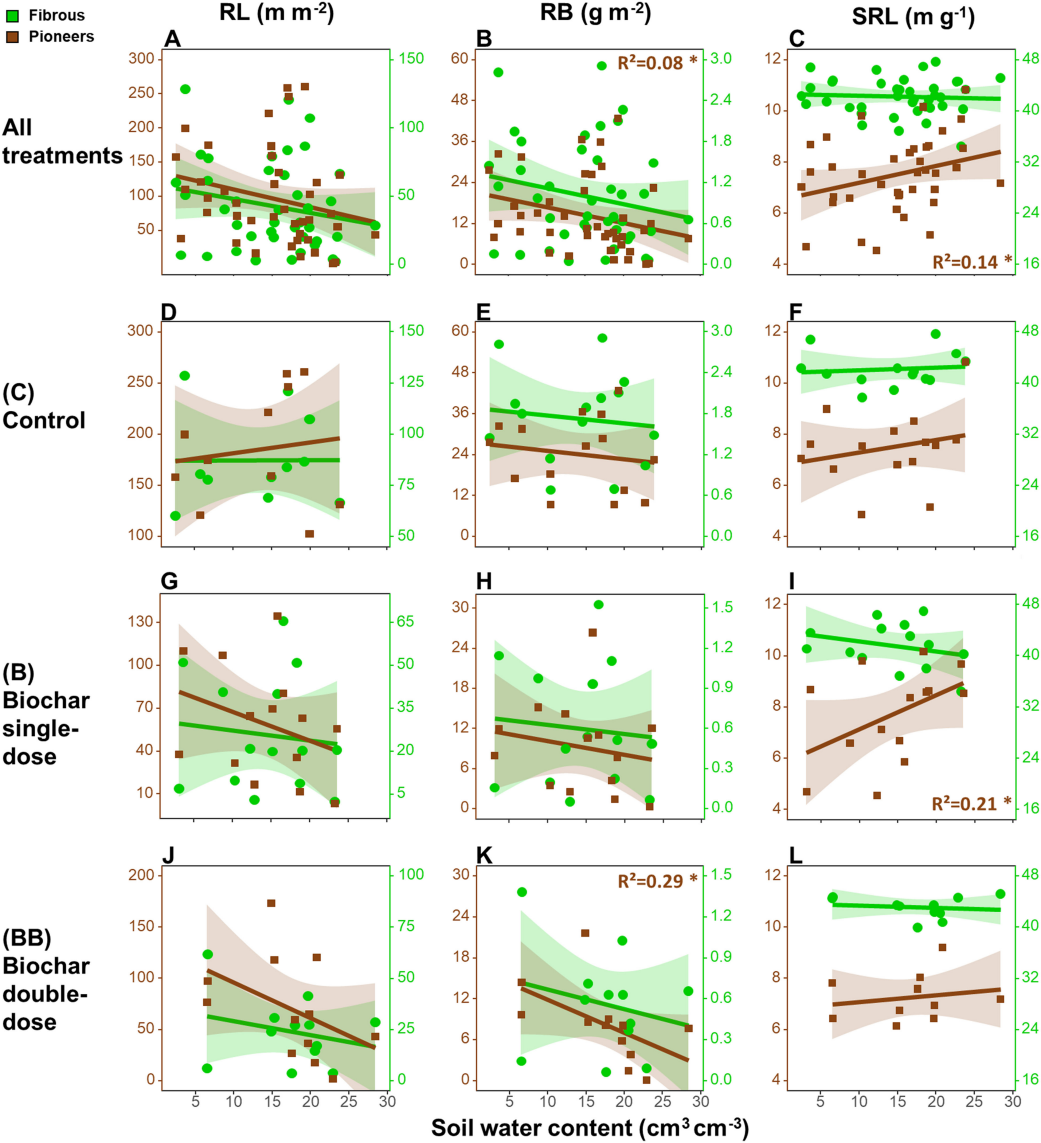
Relationships between root length (RL, **A, D, G, J**), root biomass (RB; **B, E, H, K**), specific root length (SRL; **C, F, I, L**) in the upper 20 cm soil depth and the seasonal variation of soil water content considering all treatments together **(A–C)**, or only in the control **(D–F)**, biochar single-dose **(G–I)** and double-dose **(J–L)** plots in the period between 15th May 2019 and 15th April 2020. The determination coefficient (R²) of the linear regression models is reported only when significant. Legend: *p<0.05. Number of observations in each regression = 48.

Concerning the relationships between root traits at 0-20 cm soil depth and the seasonal variation of soil water content, when all treatments were analyzed together (**Figures 1A–C**), only pioneer roots showed a decrease in RB (- 0.46 g m^-2^ per unit of soil water content) and an increase of SRL (+ 0.09 m g^-1^) along with increasing soil water content (**Figures 1B, C**). However, separating the different treatments (**Figures 1D–L**), we only found a significant decrease of pioneer roots’ RB (- 0.49 g m^-2^) along with increasing soil water content in the BB treatment (**Figure 1K**), and an opposite trend of SRL (+ 0.13 m g^-1^) in the B treatment (**Figure 1I**).

**Figure 2:**
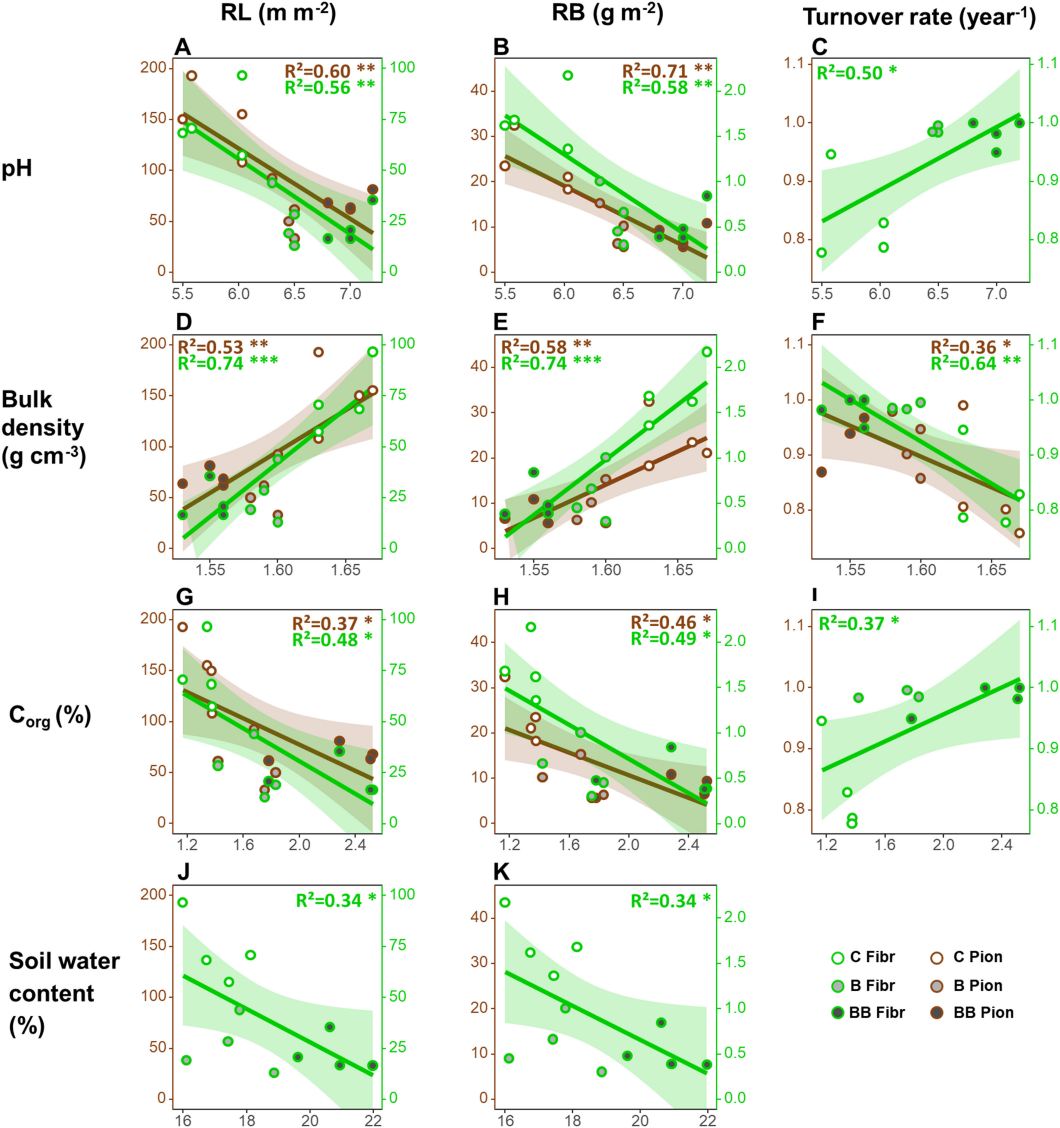
Relationships between the average values for each plot of root traits in the upper 20 cm soil depth and soil physiochemical and nutrient properties considering all treatments together. Only significant relationships are plotted, i.e. those between root length (RL, **A, D, G, J**), root biomass (RB; **B, E, H, K**), turnover rate (**C, F, I**) and soil pH **(A–C)**, bulk density **(D–F)**, C_org_ **(G–I)** and soil water content **(J, K)**. Legend: *p<0.05, **p<0.01, ***p<0.001. Number of observations in each regression = 12.

Considering the relationships between root traits at 0-20 cm soil depth and soil properties, both fibrous and pioneer roots showed a decrease in RL (respectively, - 36.9 and – 690.2 m m^-2^ per unit of soil pH) and RB (respectively, - 0.9 and – 13.3 g m^-2^) along with increasing soil pH (**Table 2**; **Figures 2A, B**). Both root types showed also a decrease in RL (respectively, - 40.2 and - 64.4 m m^-2^ per unit of soil C_org_) and RB (respectively, - 0.9 and - 12.4 g m^-2^) along with increasing C_org_ (**Table 2**; **Figures 2G, H**), but only fibrous roots showed a significant decrease in RL (- 8.1 m m^-2^ per unit of soil water content) and RB (- 0.2 g m^-2^) along with increasing soil water content (**Table 2**; **Figures 2J, K**). On the contrary, fibrous and pioneer roots showed an increase in RL (respectively, + 528.4 and + 813.0 m m^-2^ per unit of soil bulk density) and RB (respectively, + 12.2 and 147.1 g m^-2^) along with increasing soil bulk density (**Table 2**; **Figures 2D, E**). Only fibrous roots showed a significant increase in the turnover rate along with increasing soil pH and C_org_ (both being + 0.1 year^-1^), while both fibrous and pioneer roots showed a significant decrease in the turnover rate along with increasing soil bulk density (respectively, - 1.5 and -1.1 year^-1^) (**Table 2**; **Figures 2C, F, I**). The root production and SRL of both root types did not show significant relationships with all soil physicochemical and nutrient properties, as well as the CEC, N_tot_, Ka_av_, Ca_av_, Mg_av,_ and Na_av_ did not show significant relationships with any root traits (**Table 2**).

**Table 2:** Coefficients of simple linear regressions between root traits (dependent variables) and soil physicochemical and nutrient properties (independent variables) at 0-20 cm depth.

			pH	Bulk density (g cm^−3^)	Water content (%)	CEC (meq 100g^−1^)	C_org_ (%)	N_tot_ (%)	K_av_ (mg K2O kg^−1^)	Ca_av_ (mg CaO kg^−1^)	Mg_av_ (mg MgO kg^−1^)	Na{sb}{/sb}_av_ (mg Na kg^−1^)
RL (m m^−2^)	**Fibr**	**β**	**-36.9**	**528.4**	**-8.1**	-7.6	**-40.2**	-314.4	-4.3	-2.1	-2.7	-5.9
		**R²**	**0.56**	**0.74**	**0.34**	0.16	**0.48**	0.20	0.23	0.21	0.15	0.05
		**p**	**0.005**	**0.000**	**0.044**	0.197	**0.013**	0.151	0.121	0.139	0.208	0.525
	**Pion**	**β**	**-69.2**	**813.0**	-9.9	-12.8	**-64.4**	-405.2	-5.7	-2.9	-5.9	-2.5
		**R²**	**0.60**	**0.53**	0.16	0.14	**0.37**	0.10	0.12	0.12	0.23	0.00
		**p**	**0.003**	**0.007**	0.202	0.234	**0.035**	0.319	0.269	0.263	0.119	0.883
RB (g m^−2^)	**Fibr**	**β**	**-0.9**	**12.2**	**-0.2**	-0.2	**-0.9**	-7.3	-0.1	-0.0	-0.1	-0.1
		**R²**	**0.58**	**0.74**	**0.34**	0.16	**0.49**	0.20	0.23	0.20	0.16	0.04
		**p**	**0.004**	**0.000**	**0.046**	0.191	**0.012**	0.151	0.118	0.143	0.198	0.548
	**Pion**	**β**	**-13.3**	**147.1**	-1.9	-2.8	**-12.4**	-96.4	-1.3	-0.7	-1.0	-0.7
		**R²**	**0.71**	**0.58**	0.19	0.22	**0.46**	0.18	0.21	0.20	0.22	0.00
		**p**	**0.001**	**0.004**	0.151	0.12	**0.016**	0.163	0.137	0.149	0.121	0.794
SRL (m g^−1^)	**Fibr**	**β**	0.8	-5.3	0.3	0.1	1.3	13.6	0.3	0.1	0.2	0.8
		**R²**	0.04	0.01	0.08	0.00	0.10	0.07	0.20	0.08	0.19	0.14
		**p**	0.529	0.723	0.349	0.826	0.323	0.423	0.146	0.389	0.160	0.232
	**Pion**	**β**	0.4	-4.8	0.1	0.2	0.6	7.1	0.1	0.0	0.0	0.1
		**R²**	0.11	0.09	0.11	0.13	0.13	0.14	0.23	0.05	0.02	0.00
		**p**	0.289	0.353	0.291	0.260	0.255	0.229	0.114	0.471	0.701	0.783
Root Production (g m^−2^ year^−1^)	**Fibr**	**β**	-0.5	8.4	-0.2	-0.1	-0.5	-9.0	-0.1	-0.1	0.0	0.0
		**R²**	0.13	0.29	0.21	0.08	0.10	0.22	0.21	0.31	0.03	0.00
		**p**	0.280	0.009	0.158	0.412	0.356	0.142	0.151	0.075	0.586	0.995
	**Pion**	**β**	-5.9	118.4	-2.4	-1.0	-9.2	-101.1	-1.4	-0.6	0.1	-1.4
		**R²**	0.12	0.31	0.25	0.02	0.21	0.17	0.20	0.13	0.00	0.02
		**p**	0.271	0.059	0.096	0.634	0.135	0.183	0.144	0.245	0.873	0.663
Turnover rate (year^−1^)	**Fibr**	**β**	**0.1**	**-1.5**	0.0	0.0	**0.1**	1.2	0.0	0.0	-0.0	0.0
		**R²**	**0.50**	**0.64**	0.33	0.19	**0.37**	0.28	0.22	0.28	0.15	0.00
		**p**	**0.015**	**0.003**	0.066	0.184	**0.045**	0.091	0.142	0.094	0.239	0.781
	**Pion**	**β**	0.1	**-1.1**	0.0	0.0	0.1	0.8	0.0	0.0	0.0	0.0
		**R²**	0.18	**0.36**	0.18	0.03	0.15	0.14	0.17	0.20	0.00	0.00
		**p**	0.173	**0.038**	0.169	0.570	0.211	0.231	0.181	0.149	0.834	0.885

CEC, cation exchange capacity; org, organic; tot, total; av, available.

Emboldened values are those with p-value lower than 0.05. Legend: β = slope, p = p-value.

### 3.4 Root production and turnover

**Figure 3:**
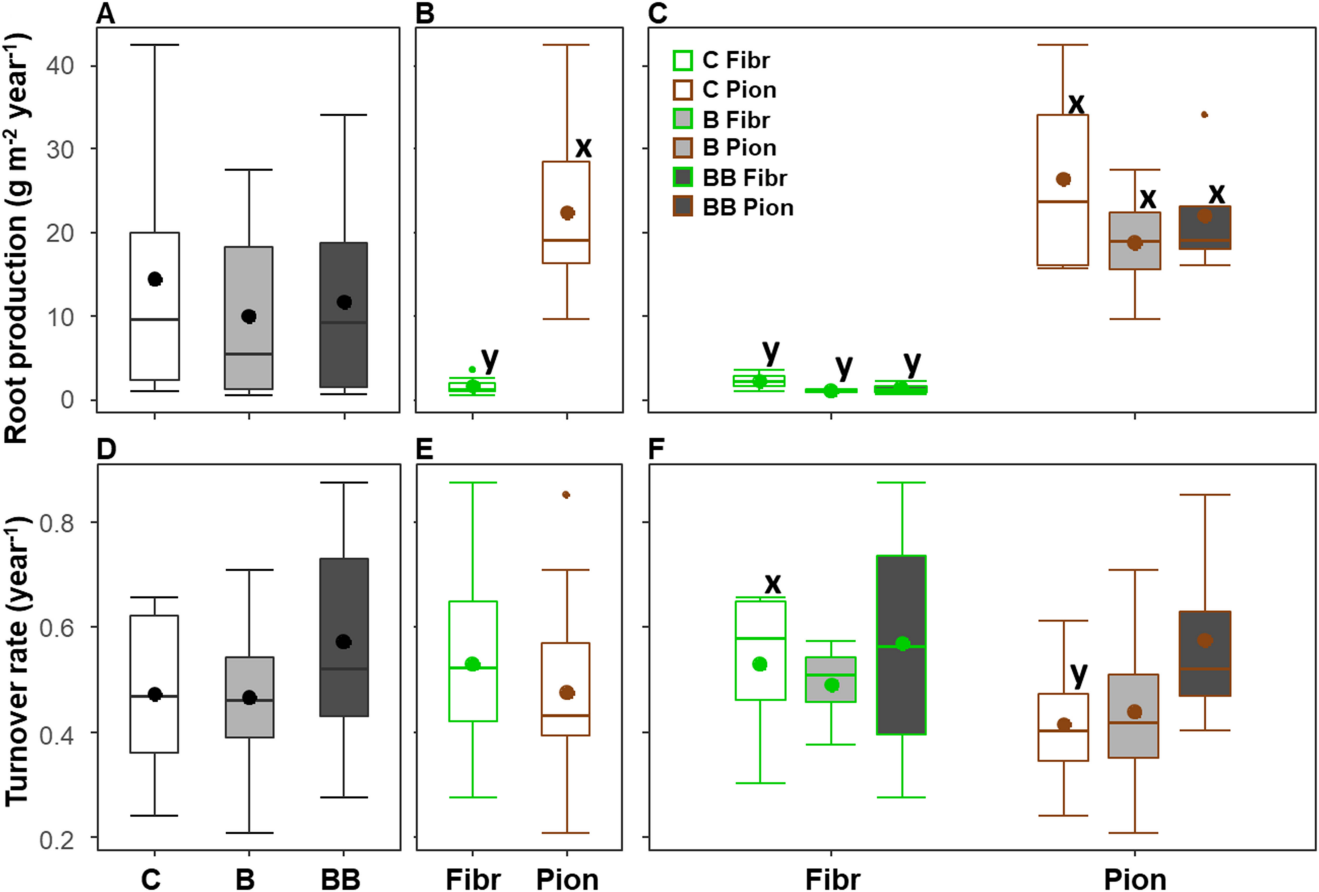
Variation of root production **(A–C)** and turnover rate **(D–F)** in the upper 40 cm soil depth among different experimental treatments (control, C; biochar single-dose, B; biochar double-dose, BB) **(A, D)**, fine root types (fibrous, Fibr; pioneers, Pion) **(B, E)**, and in relation to both treatments and fine root types **(C, F)**. The boxplot indicates the median and the mean (respectively, line and circle in the middle of the boxes), the interquartile range (boxes), 1.5 times the interquartile range (whiskers) and outliers (circle). Letters a and b indicate significant differences (p < 0.05) among control and treatments. Letters x and y indicate significant differences (p < 0.05) between fine root types. Number of observations = 24.

The root production and turnover showed an average value of respectively 12 ± 2.6 g m^-2^ year^-1^ and 0.5 ± 0.04 year^-1^ (mean ± standard error) considering the upper 40 cm soil depth, and they did not show differences among the control, B, and BB treatments (**Table 3**; **Figures 3A, D**). The root type exerted a significant influence only on the root production (**Table 3**), with fibrous roots showing lower values than pioneer ones (respectively 1.62 ± 0.3 and 22.5 ± 2.7 g m^-2^ year^-1^; **Figure 3B**). The same pattern of root production was evident also when treatments and root types were analyzed separately (**Figure 3C**), while the turnover rate was higher for fibrous roots than pioneer ones (0.53 ± 0.1 and 0.15 ± 0.1 year^-1^), but only in the control (**Figure 3F**).

**Table 3:** Three-way aligned ranks transformation ANOVA to test the effects of treatment (control, biochar single dose, biochar double dose), root type (pioneer vs. fibrous), time (four sampling points) and their interactions in the upper 40 cm soil depth, or to test the effects of treatment, root type, soil depth (0-10, 10-20, 20-30, 30-40 cm) and their interactions, on root length (RL), root biomass (RB), specific root length (SRL), root production and turnover rate.

	RL (m m^-2^)		RB (g m^-2^)		SRL (m g^-1^)		Root production (g m^-2^ year^-1^)		Turnover (year^-1^)	
	F	p	F	p	F	p	F	p	F	p
Overall (0-40 cm)										
Treatment	**6.4**	**0.019**	**7.5**	**0.012**	1.0	0.408	1.1	0.373	0.3	0.769
Root type	**150.5**	**0.000**	**276.8**	**0.000**	**220.3**	**0.000**	**141.4**	**0.000**	1.4	0.270
Date	**4.2**	**0.009**	**5.8**	**0.001**	**4.4**	**0.007**				
Treatment x root type	3.0	0.057	**20.2**	**0.000**	**3.3**	**0.045**	1.4	0.296	0.8	0.487
Treatment x date	0.3	0.911	1.1	0.348	0.2	0.977				
Root type x date	**3.1**	**0.032**	**5.2**	**0.003**	**2.8**	**0.045**				
Treatment x root type x date	0.3	0.951	1.1	0.371	0.9	0.471				
Considering 10 cm soil depth layer										
Treatment	**8.8**	**0.007**	**15.8**	**0.001**	**7.7**	**0.011**	3.6	0.007	**9.3**	**0.007**
Root type	**132.7**	**0.000**	**428.3**	**0.000**	**823.3**	**0.000**	**221.6**	**0.000**	3.3	0.076
Depth	**30.8**	**0.000**	**26.5**	**0.000**	**2.8**	**0.040**	**14.9**	**0.000**	**27.4**	**0.000**
Treatment x root type	3.0	0.051	**27.2**	**0.000**	1.8	0.169	**3.2**	**0.049**	2.8	0.068
Root type x depth	2.6	0.058	**6.9**	**0.000**	**3.8**	**0.001**	**4.5**	**0.000**	1.1	0.395
Treatment x depth	**6.2**	**0.000**	**24.7**	**0.000**	0.4	0.743	**8.7**	**0.000**	1.1	0.360
Treatment x root type x depth	0.3	0.934	**6.6**	**0.000**	**3.3**	**0.003**	**3.8**	**0.003**	1.8	0.120

Emboldened values are those with p-value lower than 0.05.

**Figure 4:**
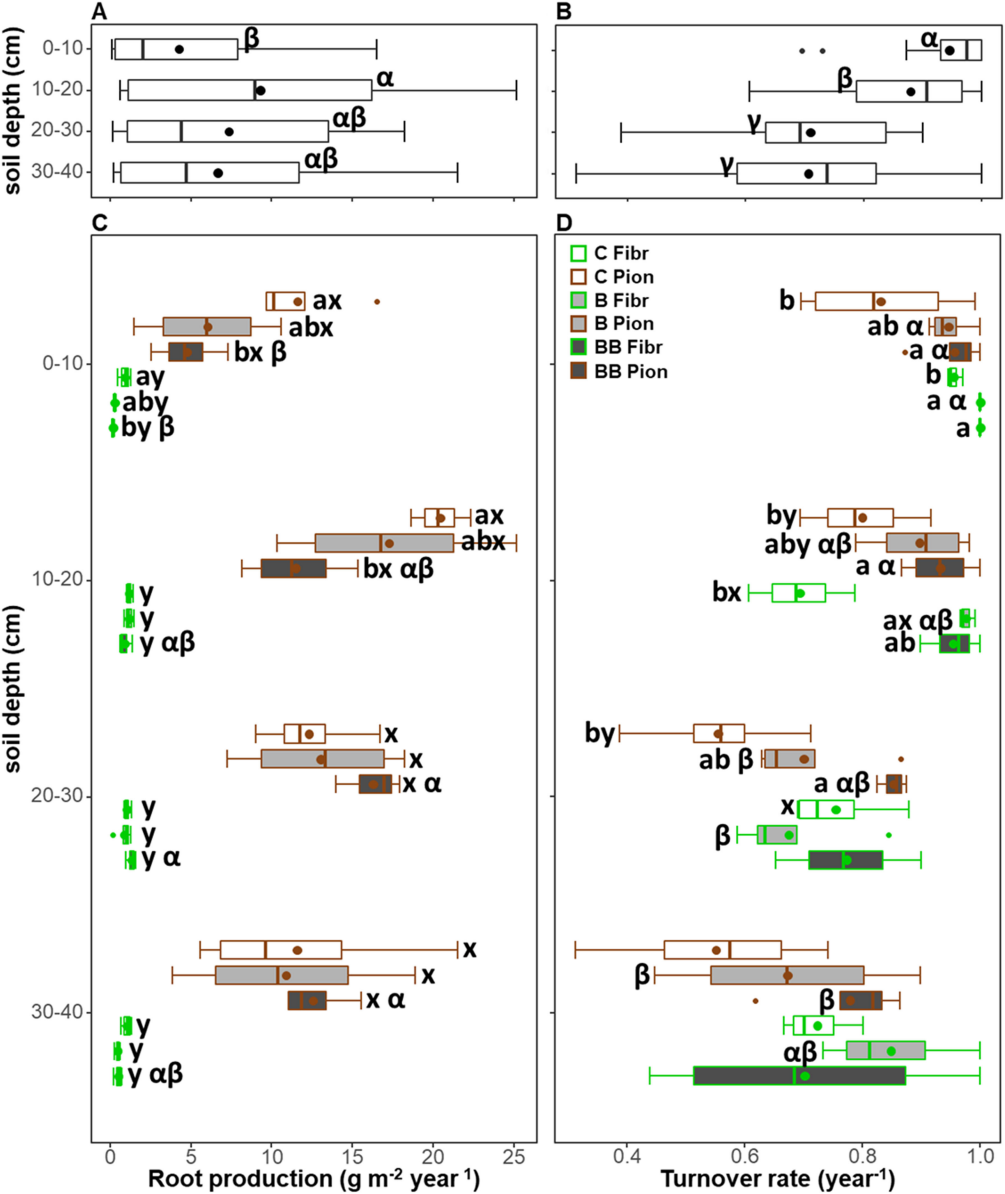
Comparison of root production **(A, C)** and turnover **(B, D)** for each 10 cm soil depth layer **(A, B)** and in relation to both treatments (control, C; biochar single-dose, B; biochar double-dose, BB) and fine root types (fibrous, Fibr; pioneers, Pion) **(C, D)** in the upper 40 cm soil depth. The boxplot indicates the median and the mean (respectively, line and circle in the middle of the boxes), the interquartile range (boxes), 1.5 times the interquartile range (whiskers) and outliers (circle). Letters a and b indicate significant differences (p < 0.05) among control and treatments. Letters x and y indicate significant differences (p < 0.05) between fine root types. Letters α and β indicate significant differences (p < 0.05) of each variable across soil depth layers. Number of observations = 96.

The soil depth exerted a significant effect on root production and turnover, in combination with treatment only for root production (**Table 3**). When both root types and treatments were analyzed together, the root production was lower in the 0-10 cm soil depth layer (4.27 ± 1.01 g m^-2^ year^-1^) with respect to the 10-20 cm (9.31 ± 1.94 g m^-2^ year^-1^), while no differences were observed with respect to the 20-30 and 30-40 cm soil depth layers (respectively being 7.37 ± 1.49 and 6.67 ± 1.45 g m^-2^ year^-1^; **Figure 4A**). On the contrary, a higher turnover rate was observed in the 0-10 and 10-20 cm soil depth layers (respectively, 0.95 ± 0.02 and 0.88 ± 0.03 year^-1^) with respect to the other layers, with higher values being those at 0-10 cm soil depth (**Figure 4B**). Differences in root production along soil depth were more marked in the BB treatment, with pioneer roots showing lower values at 0-10 cm compared to 20-30 and 30-40 cm soil depth layers, and fibrous roots showing lower values at 0-10 cm compared only to 20-30 cm soil depth layers (**Figure 4C**). However, differences in turnover rate along soil depth were significant in the BB treatment only for pioneer roots, having higher values at 0-10 and 10-20 cm compared to 30-40 cm soil depth layers (**Figure 4D**). In addition, the turnover rate showed differences along soil depth also in the B treatment for both fibrous and pioneer roots, being higher at 0-10 cm, compared to 20-30 and 30-40 cm soil depth layers for pioneer roots, or compared only to 20-30 cm soil depth for fibrous roots (**Figure 4D**).

In all soil depth layers and within each treatment, fibrous roots showed a lower root production than pioneer roots, while the turnover rate was largely comparable, with the only exceptions at the 10-20 cm soil depth layer in the B treatment and at the 20-30 cm soil depth layer in the control, where the turnover rate was higher in fibrous roots (**Figures 4C, D**).

Within the 0-10 cm soil depth layer, pioneer roots showed a higher root production and a lower turnover rate in the control compared only to the BB treatment, while fibrous roots showed a higher root production in the control compared to the BB treatment, and a lower turnover rate in the control compared to both B and BB treatments (**Figures 4C, D**). Within the 10-20 cm soil depth layer, fibrous and pioneer roots showed the same pattern observed in the soil layer above, with the only difference being that for fibrous roots the root production did not show a significant difference among control and treatments (**Figures 4C, D**). Within the 20-30 cm and 30-40 cm soil depth layers, both root production and turnover rate did not show differences among control, B, and BB treatments, except for pioneer roots at 20-30 cm soil depth having lower turnover rate in the control compared only to the BB treatment (**Figures 4C, D**).

### 3.5 Root length, biomass, and specific root length

**Figure 5:**
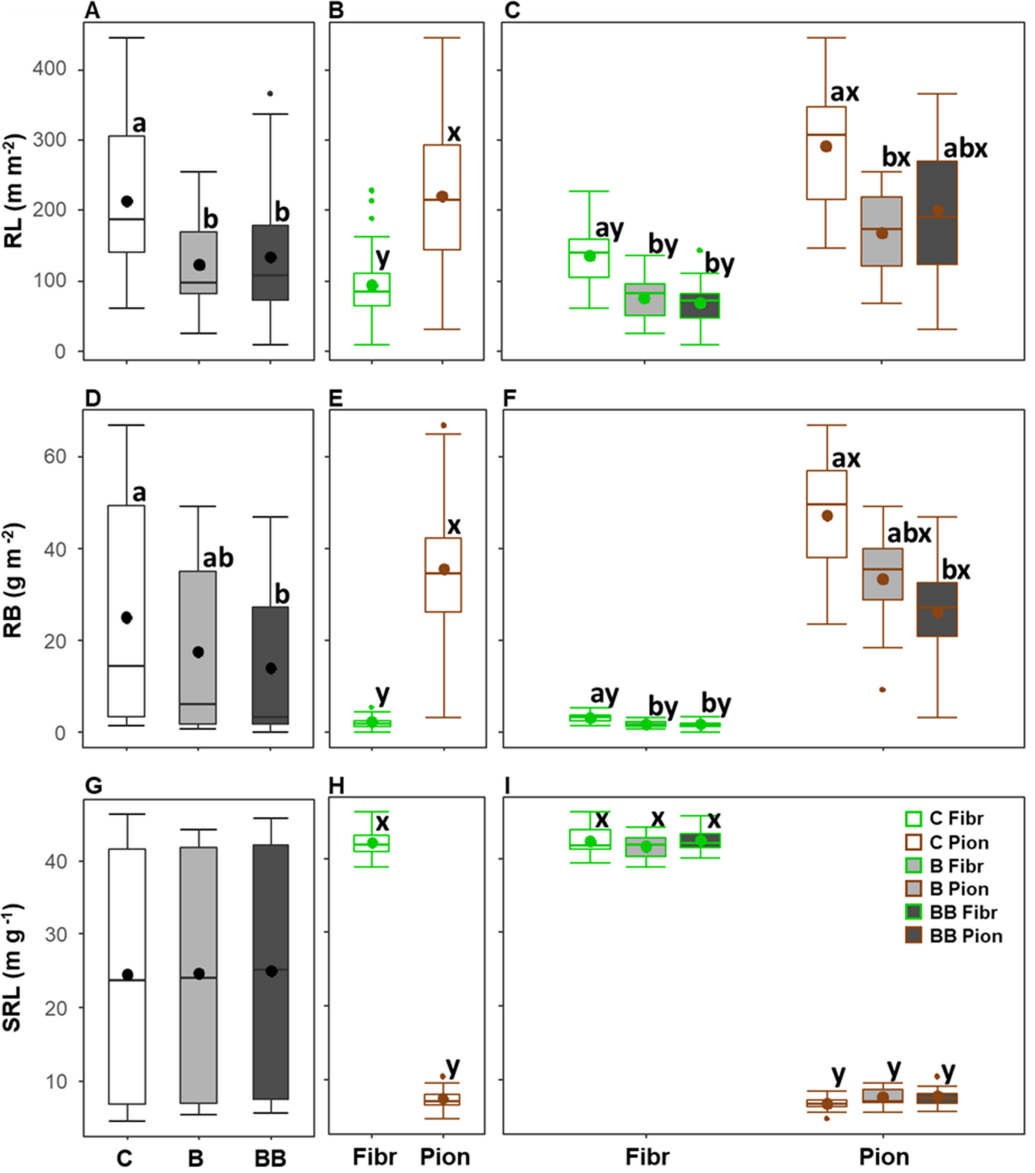
Variation of root length (RL, **A-C**), root biomass (RB; **D-F**), and specific root length (SRL; **G-I**) in the upper 40 cm soil depth among different experimental treatments (control, C; biochar single-dose, B; biochar double-dose, BB) **(A, D, G)**, fine root types (fibrous, Fibr; pioneers, Pion) **(B, E, H)**, and in relation to both treatments and fine root types **(C, F, I)**. The boxplot indicates the median and the mean (respectively, line and circle in the middle of the boxes), the interquartile range (boxes), 1.5 times the interquartile range (whiskers) and outliers (circle). Letters a and b indicate significant differences (p < 0.05) among control and treatments. Letters x and y indicate significant differences (p < 0.05) between fine root types. Number of observations = 96.

Considering pooled data in the upper 40 cm soil depth, the RL showed an average value of 156.9 ± 10.1 m m^-2^, the RB was 18.9 ± 2.0 g m^-2^, and the SRL was 24.8 ± 1.8 m g^-1^. Only the RL and RB were significantly influenced by the biochar treatments (**Table 3**), being significantly higher in the control (respectively, 213.5 ± 18.8 m m^-2^ and 25.1 ± 4.3 g m^-2^) compared to the B and BB treatments for RL (being respectively 122.7 ± 11.6 and 134.5 ± 17.2 m m^-2^; **Figure 5A**), or compared only to the BB treatment for RB (14 ± 2.6 g m^-2^; **Figure 5D**). The SRL did not show significant differences between the control and biochar treatments (**Figure 5G**). At the same time, the root type had a significant effect on all RL, RB, and SRL (**Table 3**), with fibrous roots having a lower RL (93.9 ± 7.0 m m^-2^) and RB (2.2 ± 0.2 g m^-2^), but a higher SRL (42.2 ± 0.3 m g^-1^), with respect to pioneer ones (respectively being 219.9 ± 14.0 m m^-2^, 35.6 ± 2.1 g m^-2^, and 7.3 ± 0.2 m g^-1^; **Figures 5B, E, H**). RB and SRL, as well as RL albeit marginally significant, showed a significant interaction among treatment and root type (**Table 3**), confirming the same differences among treatments even considering fibrous and pioneer roots separately (**Figures 5C, F, I**), except for the RL of pioneer roots that was not different in the BB treatment, while the RB of fibrous roots in the B treatment was lower and similar to the BB treatment (**Figures 5C, F**).

**Figure 6:**
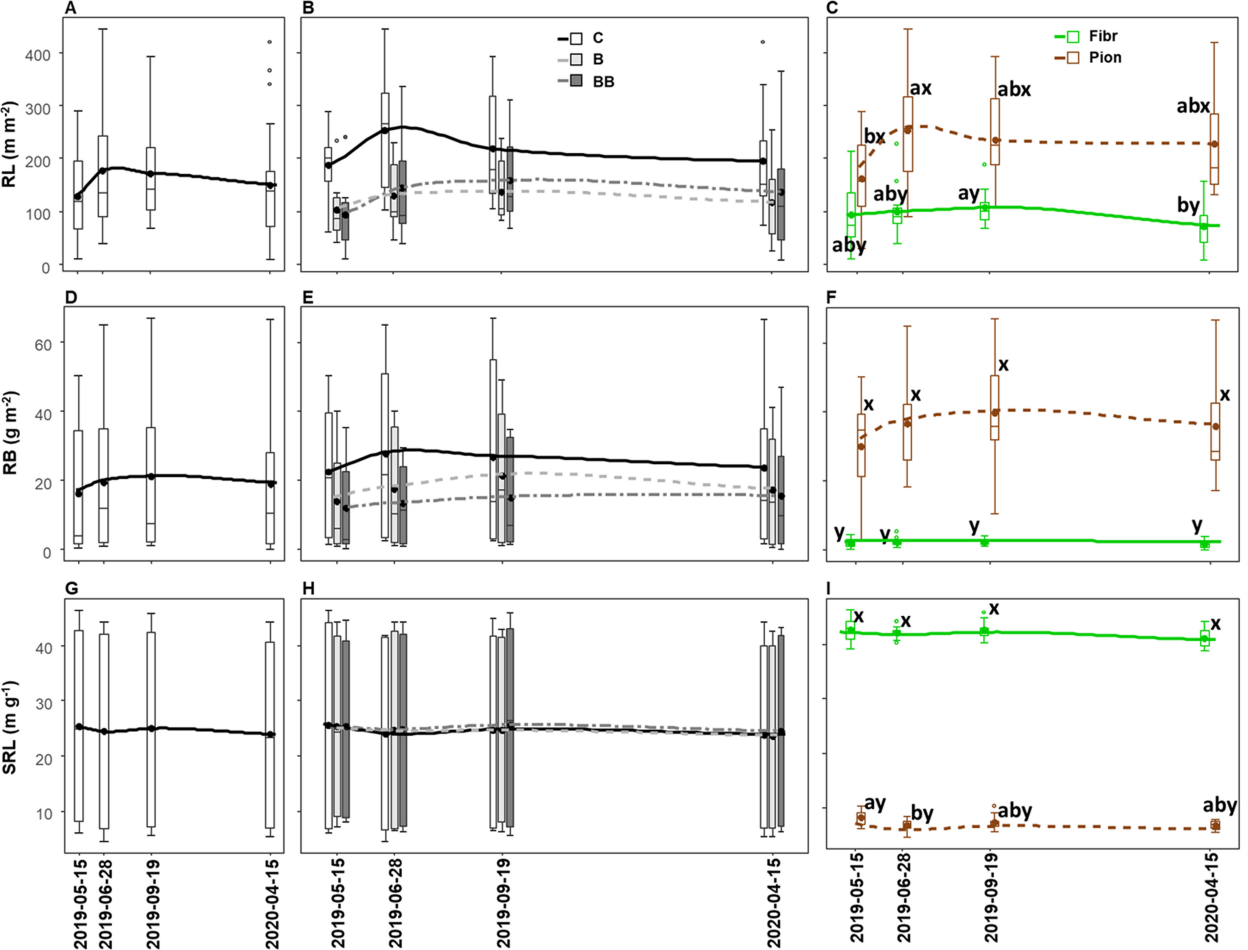
Seasonal variation of root length (RL; **A-C**), root biomass (RB; **D-F**), and specific root length (SRL; **G-I**) in the upper 40 cm soil depth in relation to sampling date **(A, D, G)**, sampling date x different experimental treatments (control, C; biochar single-dose, B; biochar double-dose, BB) **(B, E, H)** and sampling date x fine root types (fibrous, Fibr; pioneers, Pion) **(C, F, I)**. The boxplot indicates the median and the mean (respectively, line and circle in the middle of the boxes), the interquartile range (boxes), 1.5 times the interquartile range (whiskers) and outliers (circle). Letters a and b indicate significant differences (p < 0.05) among sampling dates. Letters x and y indicate significant differences (p < 0.05) between fine root types. Number of observations = 96.

The sampling date showed a significant effect on RL (**Table 3**), which increased between late spring (lowest values) and early summer (highest values), followed by a slow decrease toward lower values observed in the next year’s spring, although being not significant in the *post-hoc* comparison (**Figure 6A**). Such RL variation was more pronounced in the control than biochar treatments (**Figure 6B**), but it was significant only when root types were analyzed separately, with pioneer roots showing a higher RL value in June, contrary to fibrous ones, which were higher in September (**Figure 6C**). Despite we found an overall significant effect of sampling date on RB and SRL (**Table 3**), no statistically significant differences between the different sampling dates were observed (**Figures 6D, G**), even when looking at the seasonal pattern within the single treatment (**Figures 6E, H**) or root type, except for pioneer roots that showed higher SRL in May compared to June 2019 (**Figures 6F, I**). The differences in RL, RB, and SRL between control and treatments within the same sampling date resembled the overall variation, with RL and RB being higher in the control, although without statistical differences (**Figures 6B, E, H**), while the differences between fibrous and pioneer roots were significant throughout the whole season, with lower RL and RB, but higher SRL, for fibrous roots (**Figures 6C, F, I**).

**Figure 7:**
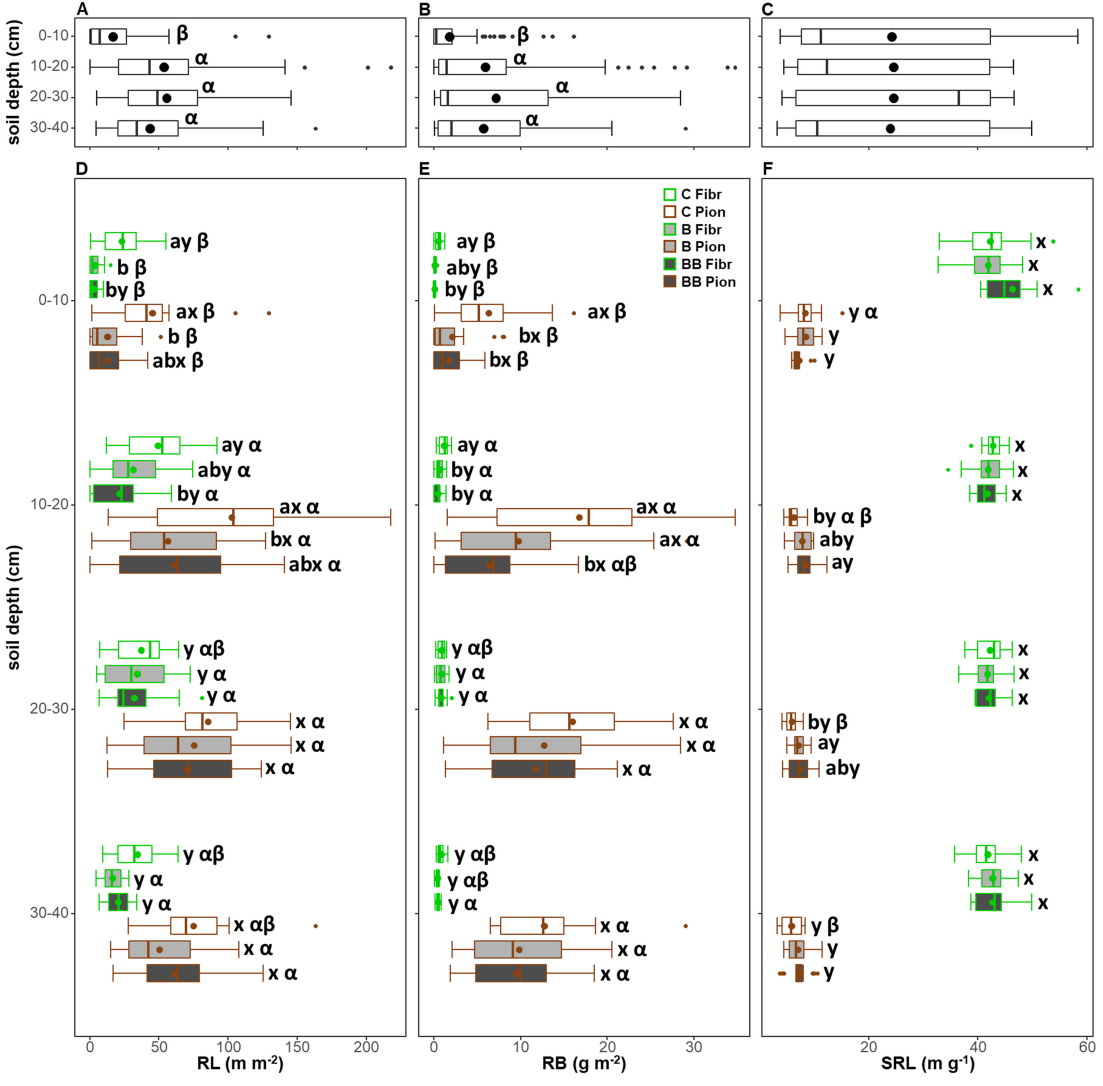
Comparison of root length (RL; **A, D**), root biomass (RB; **B, E**), and specific root length (SRL; **C, F**) for each 10 cm soil depth layer **(A-C)** and in relation to both treatments (control, C; biochar single-dose, B; biochar double-dose, BB) and fine root types (fibrous, Fibr; pioneers, Pion) **(D-F)** in the upper 40 cm soil depth. The boxplot indicates the median and the mean (respectively, line and circle in the middle of the boxes), the interquartile range (boxes), 1.5 times the interquartile range (whiskers) and outliers (circle). Letters a and b indicate significant differences (p < 0.05) among control and treatments. Letters x and y indicate significant differences (p < 0.05) between fine root types. Letters α, β, and γ indicate significant differences (p < 0.05) of each variable across soil depth layers. Number of observations = 384.

The soil depth exerted a significant effect on all RL, RB, and SRL (**Table 3**). However, when both root types and treatments were analyzed together, only the RL and RB were significantly lower in the 0-10 cm soil layer, without differences across the other soil layers (**Figures 7A, B**). Furthermore, the SRL showed no significant pattern with soil depth layers (**Figure 7C**). In all soil depth layers and within each treatment, the root type exerted a significant effect on all traits, also in combination with depth for RB and SRL (**Table 3**), with fibrous roots showing a lower RL and RB, but a higher SRL, with respect to pioneer roots (**Figures 7D–F**).

The treatment significantly influenced RL, RB, and SRL, also in combination with depth, for RL and RB (**Table 3**). Within the 0-10 cm soil depth layer, both fibrous and pioneer roots showed a higher RL and RB in the control compared to the B and BB treatments, except for RL of pioneer roots in the BB treatment or RB of fibrous ones in the B treatment, which did not show differences, and no differences were observed in the SRL (**Figures 7D–F**). Within the 10-20 cm soil depth layer, fibrous roots showed almost the same pattern observed in the soil layer above, although the RL in the BB treatment was not different, while the RB in the B treatment showed lower values and comparable to BB treatment (**Figures 7D, E**). The RL of pioneer roots also showed the same pattern of the soil layer above, while the RB of pioneer roots was higher in the B treatment and comparable to the control (**Figures 7D, E**). At this depth, pioneer roots also showed lower SRL in the control compared to the BB treatment (**Figure 7F**). Within the 20-30 cm soil depth layer, fibrous roots showed no differences among control and treatments both in RL, RB, and SRL, while pioneer roots showed lower SRL in the control with respect to the B treatment only (**Figures 7D–F**). Finally, within the 30-40 cm soil depth layer, no differences were observed between control and treatments for all root traits (**Figures 7D–F**).

## 4 Discussion

### 4.1 Soil physicochemical properties

Our results support the first part of our hypothesis (*i*) showing that the effects of the biochar addition to sandy-clay-loam soil are still detectable 10 years after its application, causing a significant amelioration of soil physicochemical properties. Biochar-treated soil still showed a lower bulk density and a higher pH, cation exchange capacity, and water content, with the highest values observed in the BB treatment. In addition, we measured a significant increment in some crucial soil nutrients, among them organic carbon, total nitrogen, available potassium, magnesium, and calcium. Similar changes in soil physiochemical properties and nutrient contents are commonly reported in studies involving the amendment of soil with biochar (Montagnoli et al., 2021; Baronti et al., 2022), highlighting a general increase in fertility of the soil and, consequently, improved plant growth and productivity (Ding et al., 2016; El-Naggar et al., 2019). Our data further corroborate this thesis, as biochar-treated plants produced significantly more fruits than control ones. Biochar can increase plant productivity, especially when applied together with fertilizers as in our experiment (Backer et al., 2017; Nan et al., 2020; Liu et al., 2021), but our results prove the effectiveness of biochar amendment also on the long term. As previously observed by Genesio et al. (2015) on a short-term analysis in 1-3 years from the biochar application, also in our study the weight of fruit was not significantly (p > 0.05) different between the B and BB treatments, confirming that a single biochar dose of 16.5 dry t ha^−1^ is enough to saturate the grapevine fruit production even in the long-term. Similarly, we observed no differences between the B and BB treatments in root production and turnover rate, RL, and RB, suggesting the presence of a long-term saturation effect also on root development. Considering the short- and long-term effects, a single dose of biochar is probably enough to obtain the desired positive effects on grapevine plants. Among the analyzed soil nutrients, only soil C_org_, as well as Ca_av_ and Mg_av_ even if only marginally significant, were higher with a single dose of biochar, highlighting their important role in grapevine fruit production (Zlámalová et al., 2015), and suggesting the importance of biochar addition to counteract calcium and magnesium deficiency, even at the long-term.

The physical analysis of the treated soil revealed a higher water content in plots amended with the double dose of biochar (BB), suggesting that grapevine plants in these plots were growing for 10 years subject to greater water availability. It could be speculated, that biochar-mediated increases in water availability could potentially favor pathogens. However, specific studies showed that the disease severity frequently exhibits a U-shaped curve of response versus biochar dose, with a minimum in disease severity observed at an intermediate biochar dose and greater disease severity at low and high doses (Graber et al., 2014). A recent study on the same vineyard showed that the double dose of biochar (33 t ha^−1^) reduced the abundance of putative plant pathogens like *Phaeoacremonium* and *Aspergillus* present in the treated soil when compared to non-treated soil (Idbella et al., 2024).

Root foraging in low soil moisture conditions is crucial for plant growth and an inverse relationship between fine root biomass and water content has been already observed both in grapevine (Bauerle et al., 2008) and different tree species (Montagnoli et al., 2012a, 2014, 2019). Grapevine plants growing with higher soil water availability have a lower need to explore a large soil volume in search of water and nutrients. In our study, we observed a similar trend with the lower RB values observed at the higher water contents and with the highest biochar dose. Furthermore, we observed a more pronounced RB and RL reduction in the 10-20 cm soil layer, both in fibrous and pioneer roots, probably due to particularly favorable growth conditions in this specific soil layer. Finally, we observed an increase in the pioneer roots’ SRL with the increase of the soil water content when pooled data at 0-20 cm soil depth were analyzed during the season, suggesting that in general with higher water availability plants favor the pioneer roots’ elongation despite the radial growth, in order to expand the root net designated to the transport of water. On the contrary fibrous roots showed no significant pattern, suggesting that no changes are done to the part of the root functioning mainly as water uptake. A similar trend was observed on very fine roots (0-0.5 mm) in *Quercus cerris* (Montagnoli et al., 2012a).

In addition to the soil water content, further soil properties were shown to have a significant effect on the grapevine’s root system. The 10-year-old biochar amendment, which increased soil pH and C_org_ while reducing BD, led to a decrease in RL, RB, and root lifespan (i.e., increased turnover rate). Thus, the improved soil conditions promoted by biochar induced greater fine root dynamism, resulting in fewer roots with a higher turnover rate. In less productive soils, such as the control (C), plants need to lengthen the fine-root system, especially enhancing the roots deputed to nutrient acquisition (Dalle Fratte et al., 2022), thus developing fibrous roots with a longer lifespan. Indeed, we found that the changes in soil pH and C_org_ affected the turnover rate only in fibrous roots.

### 4.2 Fine root production and turnover rate

In the upper 20 cm soil layer, biochar application was shown to reduce the root production only of pioneer roots, while no effect was observed on fibrous roots, partially supporting the second part of our hypothesis (ii). Plants growing in favorable conditions, due to the biochar amendment of soil, reduce the resources directed to fine root production and have, probably, higher availability of photosynthetic products to be directed to the development of other parts of the plant and for fruit production.

The fine roots turnover rate usually decreases with the increase of the diameter class (Gill and Jackson, 2000), thus, we expected to observe higher turnover rates in fibrous roots with respect to pioneer roots (Polverigiani et al., 2011), and this is in line with our findings in control plants and the upper soil layer (0-10 cm). However, this pattern changed when biochar was applied and both root types displayed a high turnover rate, with no differences between the two root types. Therefore, we may assert that in the long term, biochar application increases the dynamics of the roots that normally form the skeleton structure of the fine roots (i.e., pioneer roots).

In contrast with Amendola et al. (2017), who found in the short-term a reduction of the turnover rate in biochar-treated plants, in the present study we found an increase of both fibrous and pioneer roots’ turnover rate in the upper 20 cm of soil, supporting the second part of our multiple hypothesis (*ii*). When the resources are abundant, the maintenance costs of existing roots can be greater than the construction costs of new roots (Eissenstat and Yanai, 1997), forcing the plant to increase the turnover rate. The fact that we found differences only in the first upper 20 cm soil layer may be a consequence of the lower root dynamics with the increase in depth (Cordeiro et al., 2020). Superficial roots are considered to be the most active due to the higher availability of water and nutrients in the upper soil layers. In our study, the interplay of different agronomic practices may have played an important role in shaping the root turnover rate, favoring a greater root dynamism in the upper soil layers. The application of biochar in the first 30 cm depth may have cooperated with the application of inorganic fertilizers to the soil surface and the absence of irrigation. Finally, is important to highlight that the current findings are limited due to the relatively short observation period, consisting of 4 time-points spanning over 11 months. The authors call attention to future works and the importance of long-term observation (i.e., 2-3 consecutive years) of perennial root dynamics to obtain robust conclusions.

### 4.3 Fine root length, biomass, and specific root length

The overall reduction in fine root production observed in plants subjected to biochar treatment could explain the lower standing RL and RB. Indeed, the biochar application significantly suppressed the development in terms of RL and RB of both fibrous and pioneer roots even 10 years after the biochar application, supporting the third part of our hypothesis (*iii*). We can thus confirm the recent findings from the same vineyard experiment by Baronti et al. (2022) analyzing grapevine plants 10 years after the application of the biochar, even if they analyzed the overall data referred to fine roots (< 2 mm in diameter) and during one growing season. Grapevine plants growing in biochar-treated soil, characterized by higher water and nutrient availability, have a lower need to explore the soil environment (Baronti et al., 2022). Since both RL and RB with biochar decreased at the same magnitude, the SRL (that is the ratio between RL and RB) showed no differences among treatments. Our data are in contrast with those collected in the short term in a comparable experiment on grapevine (Amendola et al., 2017), where an increase in root biomass and diameter was observed in biochar-amended plants. These results suggest the existence of a double response to the biochar treatment depending on the time considered. In the short-term the stimulation of the fine-root biomass and radial growth can be considered as a quick and plastic response for colonizing the new favorable environment and enhancing nutrient transport; in the long-term, the suppression of RL and RB can be considered as an adaptation to the consolidated changed condition.

Surprisingly, despite the differences in the physicochemical properties of the soil observed between the two biochar treatments, only small differences were observed in RL and RB between the B and BB treatments, both in fibrous and pioneer roots, rejecting the fourth part of our hypothesis (iv). This observation further supports the observations of Genesio et al. (2015) concerning the saturation of the plant response to biochar already at a dose of 16.5 dry t ha^−1^.

In our study, the results of the fine root depth distribution analysis supported the fifth part of our hypothesis (*v*), unveiling that the main differences in RL and RB among treatments are concentrated in the upper 20 cm of soil, while barely any difference can be observed at a depth ranging from 20 to 40 cm. These findings are in line with Baronti et al. (2022), who suggested that the response of grapevine fine roots to biochar amendment occurs in the top soil layers and might be due to two concurrent factors. Firstly, biochar is applied only in the upper 30 cm of soil and, as its particles hardly migrate to deeper soil layers, the response on plant growth remains located in the soil layers where biochar was directly applied. Secondly, topsoil layers are more influenced by rainwater and naturally richer in nutrients due to periodic field fertilization (Zhu et al., 2021). For these reasons, Baronti et al. (2022) recently suggested that the response of grapevine fine roots to biochar amendment occurs in the very top soil layers.

## 5 Conclusions

For the first time, our study provides a view of the long-term effects of biochar amendment on *Vitis vinifera* fibrous and pioneer root dynamics. In particular, we demonstrated that even after 10 years biochar shows positive effects on the soil’s physicochemical properties (i.e., increase of soil organic matter, nutrient contents and water availability, and lower soil bulk density). Consequently, the fibrous and pioneer production is lowered, and the turnover rate is accelerated, resulting in a general reduction of the standing root web.

In the long term, a biochar-based strategy could represent a relevant tool for mitigating the negative effects of long drought periods and for rendering marginal and low-fertility lands available for cultivation. Therefore, these findings are relevant for implementing adaptive strategies to face the current climate change and sustainable agricultural practices that, as required in the frame of the European Union’s agricultural policy (CAP), reduce the intensive application of synthetic agrochemical additives. Finally, in the following years, it will be crucial to unveil the biological mechanisms that support the plant response even beyond the long-term biochar application.

## Data Availability

The raw data supporting the conclusions of this article will be made available by the authors, without undue reservation.
