# Supplementary material for: The long-term effect of biochar application to Vitis vinifera L. reduces fibrous and pioneer root production and increases their turnover rate in the upper soil layers
Source: Front Plant Sci. 2024 Oct 17;15:1384065. doi: 10.3389/fpls.2024.1384065 (PMC11538579; doi:10.3389/fpls.2024.1384065)
Supplement: Supplementary file 1 [file Table1.docx]

The long-term effect of biochar application to *Vitis vinifera* L. reduces fibrous and pioneer root production and increases their turnover rate in the upper soil layers

Peter Beatrice^1†*^, Michele Dalle Fratte^2†*^, Silvia Baronti^3^, Alessio Miali^1^, Lorenzo Genesio^3^, Francesco Primo Vaccari^3^, Bruno E. L. Cerabolini^2^, Antonio Montagnoli^1^

^1^ Laboratory of Environmental and Applied Botany, University of Insubria, Department of Biotechnology and Life Sciences, Varese, Italy

^2^ Unit of Plant Ecology and Phytogeography, University of Insubria, Department of Biotechnology and Life Sciences, Varese, Italy

^3^ Institute of BioEconomy, National Research Council, Florence, Italy

**^†^** **First authorship:** these authors share first authorship

*** Correspondence:**

Peter Beatrice: [peter.beatrice@uninsubria.it](mailto:peter.beatrice@uninsubria.it)

Michele Dalle Fratte: [michele.dallefratte@gmail.com](mailto:michele.dallefratte@gmail.com)

Supplementary material

**Supplementary table 1.** The soil's physical and chemical properties were measured by diverse studies conducted on the same vineyard after the biochar amendment in 2009 and 2010. The different experimental treatments were: control (C), biochar single-dose (B), and biochar double-dose (BB). Letters a, b, and c indicate significant differences (p < 0.05) between the soil treatments.

| **Source** | **Sampling  date** | **Bulk density (g/cm3)** | | | **pH** | | | **Water content (%)** | | | **Organic carbon (%)** | | |
| --- | --- | --- | --- | --- | --- | --- | --- | --- | --- | --- | --- | --- | --- |
|  |  | **C** | **B** | **BB** | **C** | **B** | **BB** | **C** | **B** | **BB** | **C** | **B** | **BB** |
| Maienza et al., 2017 | 2010 |  |  |  | 5,5 **b** | 5,94 **b** | 7,18 **a** |  |  |  |  |  |  |
|  |  |  |  |  | ± 0,22 SE | ± 0,24 SE | ± 0,11 SE |  |  |  |  |  |  |
| Baronti et al., 2014 | 2011 | 1,45 **a** | 1,43 **a** | 1,38 **b** |  |  |  | 12,00 **a** | 13,00 **b** | 18,00 **b** |  |  |  |
|  |  | ± 0,02 SE |  |  |  |  |  | ± 1 SE | ± 1 SE | ± 3 SE |  |  |  |
| Maienza et al., 2017 | 2013 |  |  |  | 5,24 **b** | 6,35 **a** | 6,55 **a** |  |  |  |  |  |  |
|  |  |  |  |  | ± 0,14 SE | ± 0,02 SE | ± 0,25 SE |  |  |  |  |  |  |
| Maienza et al., 2017 | 2014 |  |  |  | 5,94 **b** | 6,54 **a** | 7,31 **a** |  |  |  |  |  |  |
|  |  |  |  |  | ± 0,14 SE | ± 0,13 SE | ± 0,08 SE |  |  |  |  |  |  |
| Giagnoni et al., 2019 | 2017 |  |  |  | 5,23 **a** | 6,01 **a** | 6,21 **a** |  |  |  | 2,23 **a** | 2,56 **b** | 2,71 **b** |
|  |  |  |  |  | ± 0,14 NA | ± 0,02 NA | ± 0,15 NA |  |  |  | ± 0,06 NA | ± 0,07 NA | ± 0,09 NA |
| Idbella et al., 2024 | 2019 | 1,63 **a** | 1,59 **b** | 1,53 **c** | 6,33 **c** | 6,83 **b** | 7,07 **a** |  |  |  | 1,27 **c** | 1,73 **b** | 2,31 **a** |
|  |  | ± 0,03 SD | ± 0,02 SD | ± 0,02 SD | ± 0,06 SD | ± 0,11 SD | ± 0,10 SD |  |  |  | ± 0,07 SD | ± 0,11 SD | ± 0,12 SD |
| This study | 2020 | 1,64 **a** | 1,59 **b** | 1,55 **c** | 5,83 **c** | 6,41 **b** | 6,98 **a** | 17,13 **b** | 17,50 **b** | 20,88 **a** | 1,27 **c** | 1,73 **b** | 2,31 **a** |
|  |  | ± 0,01 SE | ± 0,00 SE | ± 0,01 SE | ± 0,12 SE | ± 0,05 SE | ± 0,07 SE | ± 0,36 SE | ± 0,44 SE | ± 0,39 SE | ± 0,06 SE | ± 0,09 SE | ± 0,14 SE |

Abbreviations: SE (standard error), SD (standard deviation), NA (not available).
